# Quantitative trait loci analysis for leg weakness-related traits in a Duroc × Pietrain crossbred population

**DOI:** 10.1186/1297-9686-43-13

**Published:** 2011-03-20

**Authors:** Watchara Laenoi, Muhammad Jasim Uddin, Mehmet Ulas Cinar, Christine Große-Brinkhaus, Dawit Tesfaye, Elisabeth Jonas, Armin M Scholz, Ernst Tholen, Christian Looft, Klaus Wimmers, Chirawath Phatsara, Heinz Juengst, Helga Sauerwein, Manfred Mielenz, Karl Schellander

**Affiliations:** 1 Institute of Animal Science, University of Bonn, Endenicher Allee 15, 53115 Bonn, Germany; 2 Reprogen, University of Sydney, 425 Werombi Road, Camden NSW 2570, Australia; 3 Livestock Center of the Veterinary Faculty, Ludwig-Maximilians University of Munich, Sankt Hubertusstrasse 12, 85764 Oberschleissheim, Germany; 4 Leibniz Institute of Farm Animal Biology, Wilhelm-Stahl-Allee 2, 18196 Dummerstorf, Germany; 5 Department of Animal and Aquatic Science, Faculty of Agriculture, Chiang Mai University, Chiang Mai, Thailand

## Abstract

**Background:**

Leg weakness issues are a great concern for the pig breeding industry, especially with regard to animal welfare. Traits associated with leg weakness are partly influenced by the genetic background of the animals but the genetic basis of these traits is not yet fully understood. The aim of this study was to identify quantitative trait loci (QTL) affecting leg weakness in pigs.

**Methods:**

Three hundred and ten F_2 _pigs from a Duroc × Pietrain resource population were genotyped using 82 genetic markers. Front and rear legs and feet scores were based on the standard scoring system. Osteochondrosis lesions were examined histologically at the head and the condylus medialis of the left femur and humerus. Bone mineral density, bone mineral content and bone mineral area were measured in the whole ulna and radius bones using dual energy X-ray absorptiometry. A line-cross model was applied to determine QTL regions associated with leg weakness using the QTL Express software.

**Results:**

Eleven QTL affecting leg weakness were identified on eight autosomes. All QTL reached the 5% chromosome-wide significance level. Three QTL were associated with osteochondrosis on the humerus end, two with the fore feet score and two with the rear leg score. QTL on SSC2 and SSC3 influencing bone mineral content and bone mineral density, respectively, reached the 5% genome-wide significance level.

**Conclusions:**

Our results confirm previous studies and provide information on new QTL associated with leg weakness in pigs. These results contribute towards a better understanding of the genetic background of leg weakness in pigs.

## Background

Leg weakness (LW) has a great impact on fitness and longevity of animals, which influences not only animal welfare but also production and reproduction performance. It has been shown that between 20 and 50% of boars completing performance tests are rejected as breeding animals because of LW problems [1]. Genetic correlations between LW-related traits and longevity in breeding sows have been reported and suggest that a better leg status would decrease involuntary culling [2,3]. Heritability estimates have been reported for LW in Duroc, Landrace, and Yorkshire sires i.e. 0.23, 0.30 and 0.39, respectively [4], and for an overall leg score in Landrace and Large White sows, i.e. 0.27 and 0.38, respectively [2]. In addition, osteochondrosis (OC) is regarded as the main cause of LW in pig [5,6]. OC is a skeletal disease characterized by disturbed bone formation, cartilage retention, or necrosis of the cartilage canal in articular cartilage [7,8] and results in economic losses mainly due to the culling of pigs in the breeding industry [9]. The disease occurs at high frequencies in growing pigs in all commercial breeds [10]. The estimated heritability of OC ranges from 0.06 to 0.5 [2,5,11,12] in different pig breeds. Moreover, OC is reported to have negative effects on important performance traits such as sow longevity, growth and feed conversion rate [12,13].

In addition to OC, bone mineral density (BMD) is generally regarded as an important parameter to assess bone growth and is associated with bone fracture risk and structural soundness in pigs. Studies in humans have shown that variation in BMD can be explained by genetic factors [14,15]. Taken together, all the data reported so far imply that LW-related traits have a low to moderate heritability. Nevertheless, genetic studies of LW-related traits in growing and finishing pigs are limited. A significant number of QTL for performance traits has been reported in pigs [16] but few studies have been devoted to LW-related traits [17-21]. Therefore, the aim of this study was to investigate QTL for LW-related traits, including leg and feet scores, OC and bone mineral traits in a Duroc × Pietrain resource population.

## Methods

### Experimental animal population

In this study, we used 310 F_2 _pigs from a Duroc × Pietrain resource population comprising three generations, Parent (P), F_1_, and F_2 _pigs, and which had been previously analysed to detect QTL for growth, carcass and meat quality traits [22]. The F_2 _pigs were generated by mating six F_1 _males with 25 F_1 _females. All animals were maintained at the Frankenforst experimental research farm at the University of Bonn. Piglets were weaned at 28 days of age, males were castrated prior to weaning and placed in pens in the post-weaning unit until 10 weeks of age. The F_2 _pigs were given an *ad libitum *diet during the whole test period and were slaughtered at approximately 105 kg live weight at around 25-26 weeks of age in the slaughterhouse of the research farm Schwarzenau in Bavaria, Germany. Tissue samples from the tail were collected within the first week after birth for DNA isolation.

### Phenotyping

Before slaughter, legs and feet were scored by the same person, using the criteria listed in additional file 1, Table S1 as guidelines to make assessments. The traits were recorded according to the rules of German performance stations [23]. Each 'leg score' is an assessment of the strength and straightness of the legs and of the stability of the joints. Leg scores ranged from 1 to 5, the optimum level being 3. Data were then transformed into a desirability scale, by using the absolute value of the original scores after subtracting three scores (score 3 becomes 0 for optimum leg score, score 2 and 4 become 1 for moderate leg score, and score 1 and 5 become 2 for poor leg score). For feet, the angle and strength of feet/leg attachment, soundness of toes and weight distribution on toes were assessed and given a score value between 1 (poor) and 3 (good). Leg and feet scores were measured on pigs walking on a solid concrete floor. After slaughter, the left fore and rear legs were removed from the carcass to carry out histological examinations of OC lesions. As OC is a bilaterally symmetrical syndrome, it was decided to examine only the left legs. The recorded OC lesions were scored 1 to 4, 4 for normal and 3 to 1 for mildly to severely affected (Additional file 1, Figure 1). OC lesions were evaluated on the head of the humerus (HH), condylus medialis humeri (CMH), head of the femur (HF) and the condylus medialis femoris (CMF). The histological examination assessed cartilage thickness, cartilage degradation and the vessel structure of cartilage canals. The histological procedures that were used have been described by Laenoi et al. [24]. The number of animals with OC on different joints ranged between 274 and 279 (Table 1). A total of 1,108 samples (532 from castrated and 576 from female pigs) out of 1,240 samples were phenotyped. In addition, the whole ulna and radius bones from the left carcass were stripped of all surrounding tissues and the bone mineral-related traits (BMD, BMC and BMA) were examined using dual energy X-ray absorptiometry (DXA) [25]. In total 275 animals were phenotyped for the DXA traits (Table 2).

**Figure 1:**
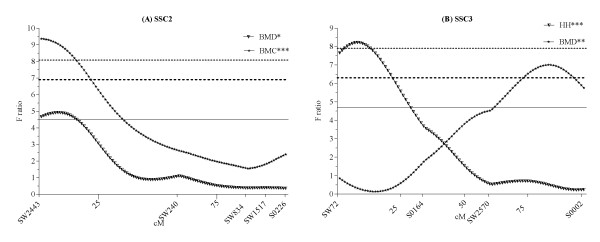
**Evidence of QTL for leg weakness related traits on SSC 2 and 3**. Marker positions along each chromosome are indicated in cM on the x-axis, and *F *values are given along the y-axis; 5% chromosome-wide (dotted line), and 1% chromosome-wide (solid line)

**Table 1:** Statistics of LW-related traits and phenotypic correlations between traits

**Traits**^**1**^	N	Mean	SD	Min	Max	Phenotypic correlation with traits							
						
						FLS	RLS	FFS	RFS	HH	CMHM	HF	CMF
FLS	310	2.65	1.08	1	5		0.28	0.23	0.22	0.08	-0.02	0.02	-0.04
RLS	310	2.70	0.66	1	5			0.23	0.19	-0.08	-0.003	-0.003	-0.05
FFS	310	2.02	0.45	1	3				0.44	-0.08	-0.12	0.01	-0.07
RFS	310	2.53	0.53	1	3					-0.01	-0.06	0.04	-0.13
HH	278	1.78	0.78	1	4						0.12	0.07	-0.007
CMH	279	1.82	0.95	1	4							0.11	-0.07
HF	274	1.98	0.84	1	4								-0.10
CMF	277	2.59	1.09	1	4								

^1^FLS = fore leg score; RLS = rear leg score; FFS = fore feet score; RFS = rear feet score; HH = OC score at head of humerus; CMH = OC score at condylus medialis humeri; HF = score OC at head of femur; CMF = score OC at condylus medialis femori

**Table 2:** Statistics of DXA phenotypes

**Traits**^**1**^	Total (n = 275)			Females (n = 145)			Castrated males (n = 130)		
	
	Mean ± SD	Min	Max	Mean ± SD	Min	Max	Mean ± SD	Min	Max
BMD (g/cm^2^)	0.96 ± 0.08	0.69	1.25	0.95 ± 0.07	0.79	1.172	0.96 ± 0.09	0.69	1.25
BMC (g)	66.72 ± 7.07	45.53	87.36	66.38 ± 6.03	45.53	83.29	67.02 ± 7.69	48.42	87.36
BMA (cm^2^)	69.67 ± 5.26	55.91	84.64	69.75 ± 5.22	55.91	84.64	69.62 ± 5.36	57.36	83.29

^1^BMD = bone mineral density, BMC = bone mineral content, BMA = bone mineral area, n = number of animals

### Genotyping

Markers used for genotyping were mainly selected from the USDA/MARC map (http://www.marc.usda.gov) and included 79 microsatellites and three biallelic markers. Marker order and genetic distances between markers are described in additional file 2, Table S2. Genotyping, electrophoresis, and allele determination were carried out with a LI-COR 4200 Automated Sequencer (DNA Analyzer, GENE reader 4200). Allele and genotyping errors were checked using Pedcheck software (v 1.1) [26]. In addition to the microsatellite markers, SNP in genes assumed to affect cartilage quality were included, i.e. SNP located in the COL10A1 and MMP3 genes. Sequences were obtained from GenBank (accession no AF222861 and FJ788664 for porcine COL10A1 and MMP3, respectively) and assays were designed to permit genotyping using a multiplex SNP genotyping platform (Beckman Coulter). The relative positions of the markers were assigned using the build, two-point and fixed options of CRIMAP software, version 2.4 [27]. Recombination units were converted into map distances using the Kosambi mapping function. Marker information content and segregation distortion were tested. A linkage map was constructed with a total length of 2588.7 cM and an average marker interval of 31.57 cM.

### Statistical analysis

The data were analysed using the software package SAS^® ^(v 9.2, SAS^® ^Inc., CA, USA). Generalized linear models (PROC GLM) were used to identify the effects of sire, dam, age, sex, birth weight, daily weight gain, litter size, litter effect, parity, season, and of carcass weight and length at slaughter on the investigated traits (Additional file 3, Table S3).

F_2 _QTL interval mapping was performed using the web-based program QTL Express [28] available at http://qtl.cap.ed.ac.uk/. The basic QTL regression model used in the present study was:

where: y_i _= phenotype of the i^th ^offspring; μ = overall mean; F_i _= fixed effect of litter; β = regression coefficient on the covariate; cov_i _= covariate of average daily gain for leg and feet score age for OC, and slaughter weight and carcass length for DXA; c_ai _= additive coefficient of the i^th ^individual at a putative QTL; c_di _= dominance coefficient of the i^th ^individual at a putative QTL; a = additive effect of the putative QTL; d = dominance effect of the putative QTL; and ε_i _= residual error.

The regression model was fitted at 1-cM intervals along each chromosome and the F-value for the QTL effect was calculated at each point. Thresholds for chromosome-wide significance were determined by 1000 data permutations [29] for individual chromosomes. Significance at the 5% chromosome-wide (CW) level was considered suggestive, 1% CW was considered significant and significance at the 5% genome-wide (GW) level as highly significant. To derive GW significance levels from the chromosome-wide significance levels, the Bonferoni correction was applied [30]. Empirical 95% confidence intervals (CI) and flanking markers for estimated QTL positions were obtained by applying the bootstrap approach with 1000 re-samplings [27]. The percentage of phenotype variation explained by a QTL was calculated as:

where, MS_R _is the mean square of the reduced model without QTL effects and MS_F _is the mean square of the full model.

## Results

### Distributions and correlation of the traits

Descriptive statistics of LW-related traits are given in Tables 1 and 2. It is important to note that in this study the direction of a desirable score is the difference between leg and feet scores and OC scores. For leg score, a low value is desirable but for feet and OC scores a high value is desirable. A high percentage of animals showed moderate fore feet scores (FFS) (79.4%) and good rear feet scores (RFS) (54.5%). Only 9.0% and 1.3% of animals showed poor feet scores for fore and rear feet, respectively. For the fore leg score (FLS), 42.3% of animals had a score value of 2 and for the rear leg score (RLS), 54.8% of animals had a score value of 3. Few animals had very poor leg scores (4.8% for fore leg and 0.3% for rear leg). Phenotypic correlations among FLS, RLS, FFS and RFS were low to medium, ranging from 0.19 to 0.44 (Table 1). The percentage of severe OC lesions in the 1,108 cartilage samples was higher in the CMF of the knee joint compared to other joints. The CMH and HH of fore limbs had healthier scores than CMF and HF. Phenotypic correlations among OC scores were very low, ranging from -0.13 to 0.12 (Table 1). BMD and BMC were not significantly different between castrated male pigs and female pigs (Table 2). The phenotypic correlation between BMD and BMC was positive (r = 0.70, *P *< 0.01). Parity, carcass length, weight at slaughter, age and average daily gain had significant (P < 0.05) effects on the measured traits (Table S2). Parity, carcass length and average daily gain had significant (P < 0.05) effects on FLS but only average daily gain (ADG) had an effect on RLS. Parity showed effects on FFS, HH, CMH and HF. Age also had an effect on HF. Parity, carcass length and weight at slaughter affected all DXA traits. BMD and BMC were highly correlated (*P *< 0.01) with the animals' weight at slaughter (r = 0.54 and 0.71, respectively).

### QTL for leg weakness-related traits

The results of the QTL analysis are given in Table 3. Eleven QTL were identified for LW-related traits on eight autosomes. Most QTL had highly significant dominance effects and three QTL were additive. Two chromosomal regions were identified for FFS (*P *≤ 0.05, CW), at 166 cM on SSC1 and at 36 cM on SSC16. Two QTL, at 87 cM (*P *≤ 0.05, CW) on SSC6 and at 26 cM on SSC18, were identified for RLS. No QTL was found for rear feet score and fore leg score. QTL associated with OC were located on SSC2, 3, 6, 10 and 14. The OC score of HH was influenced by three QTL regions, on SSC2, 3, and 6 at 14, 13 and 61 cM, respectively. A QTL for CMH was identified at 0 cM on SSC14. One QTL affecting OC score of CMF was identified on SSC10 at 70 cM. However, no suggestive QTL was found for OC score of HF. Two QTL were identified for bone mineral-related traits, one for BMD and one for BMC. A QTL for BMD was found on SSC3 at 71 cM. Only one QTL was detected for BMC, at 0 cM on SSC2. Both QTL for BMD and BMC reached a 5% GW significance.

**Table 3:** Summary of QTL detected for LW-related traits that exceed suggestive linkage

**SSC**^**a**^	**Trait**^**b**^	**POS**^**c**^	**F**^**d**^	**a ± se**^**e**^	**d ± se**^**f**^	**Var%**^**g**^	**CI95**^**h**^	**Closest markers**^**i**^
1	FFS	166	5.06*	-0.15 ± 0.05	0.16 ± 0.09	4.38	35.0 - 206.5	S0155
2	BMC	0	7.65**	-2.17 ± 0.61	3.72 ± 1.97	6.82	0.0 - 92.5	SW2443
2	HH	14	4.75*	0.39 ± 0.13	-0.59 ± 0.48	4.25	0.0 - 103.0	SW2443
3	BMD	71	6.77**	-0.04 ± 0.01	-0.02 ± 0.02	6.09	0.0 - 95.5	SW2570-S0002
3	HH	13	6.17*	-0.04 ± 0.11	0.70 ± 0.21	5.45	0.0 - 69.5	SW72-S0164
6	RLS	87	5.82*	0.09 ± 0.06	0.36 ± 0.12	5.00	27.0 - 147.5	SW193
6	HH	61	5.49*	-0.23 ± 0.09	-0.39 ± 0.18	4.88	29.0 - 150.0	S0087
10	CMF	70	5.15*	0.41 ± 0.13	0.05 ± 0.18	4.61	8.5 - 97.0	S0070
14	CMH	0	6.36*	-0.26 ± 0.09	-0.34 ± 0.14	5.56	0.0 - 43.0	SW857
16	FFS	36	6.01*	0.18 ± 0.09	0.85 ± 0.29	5.16	16.5 - 146.0	SW857
18	RLS	26	4.84*	-0.37 ± 0.12	-0.11 ± 0.36	4.20	0.0 - 112.0	SW1023-SW787

^a ^*Sus scrofa *chromosome; ^b^trait abbreviations: FLS = fore leg score, RLS = rear leg score, FFS = fore feet score, RFS = rear feet score, HH = OC score at the head of the humerus, CMH = OC score at condylus medialis humeri, HF = OC score at the head of the femur, CMF = OC score at condylus medialis femori, BMD = bone mineral density, BMC = bone mineral contents; ^c^chromosomal position in Kosambi cM; ^d^significance of the QTL: * significant on a chromosome-wide level with *P *≤ 0.05; ** significant on a chromosome-wide level with *P *≤ 0.01; *** significant on a genome-wide level with *P *≤ 0.05; ^e^additive effect and standard error: positive values indicate that Duroc alleles result in higher values than Pietrain alleles; negative values indicate that Duroc alleles result in lower values than Pietrain alleles; ^f^dominance effect and standard error; ^g^percentage of phenotypic variance explained by the QTL; ^h^95% confidence interval; ^i^closest marker to the QTL peak

In this study, most of the detected QTL appeared to have effects on only one trait, showing no effects on other traits. However, some chromosomal regions influenced more than one trait, notably on SSC2, 3 and 6.

## Discussion

In this study, we evaluated conformation traits describing leg and feet condition, osteochondrosis score and bone mineral density, which are important in selection to reduce the risk of leg weakness in pigs. However, the genetics of LW-related traits is complex [12,31]. A number of factors are known to influence the development of LW, such as nutrition imbalance, high body weight, rapid growth rate, bone and joint diseases, bad body and leg structure, and mechanical stress [11,13]. Moreover, it has been reported that the degree of LW and OC may be related to the breed and sex of animals [32]. However, in our study there was no effect of gender on LW-related traits, which implies that frequencies of LW and OC vary and depend on the genetic background of the animals [33]. It has been reported that the Duroc pure breed shows the highest incidence of OC compared to other European pig breeds (Pietrain, Landrace and Yorkshire) [32]. Our data suggest that the unfavourable QTL allele for OC originates from both Duroc and Pietrain breeds (i.e. two QTL originated from the Duroc and three from the Pietrain) (Table 3) and that in Duroc and Pietrain crossbred animals, the fore legs are less susceptible to OC than the rear legs. Andersson-Eklund et al. [17] have also reported lower OC incidences in the humerus than in the femur in a Wild boar × Large White population. In addition, our data show that the frequency of OC is high (31.05%) at CMF, which agrees with a previous report of 30.0% by Kadarmideen et al. [12].

QTL analyses for leg weakness and bone-related traits have been performed in different pig breeds, including Landrace purebred [34], White Duroc × Erhualian [19,21], Large White × Meishan [20], Duroc × Landrace and Duroc × Large White crossbred [18], and Wild boar × Large White [17]. To the best of our knowledge, our study is the first to map QTL for LW-related traits in a Duroc and Pietrain intercross. We have identified 11 QTL some of which being novel and some confirming previous studies [17-21,34], as described in the next section. However, large confidence regions were obtained in this experiment, which represents a common problem in QTL studies and hampers the comparison of QTL results and their interpretation in terms of causative genes, since large confidence intervals can contain many potential candidate genes [35].

In this study, a QTL for FFS was detected on SSC1 at 166 cM. QTL for the same trait have been reported at 89 cM in a Landrace purebred [34] and at 52 cM in a Large White × Meishan intercross [20] on the same chromosome. The dominant QTL for FFS found on SSC16 at 36 cM is close to a previously reported dominant QTL at 27 cM for rear leg score [19]. The QTL identified for RLS on SSC6 and SSC18 are new and do not overlap with any previous study. A QTL associated with rear leg score was observed on SSC6, close to marker *SW193 *(SSC6q2.1), where the gene for transforming growth factor-beta 1 (*TGFβ1*) is located [36]. This gene is an important candidate for LW-related traits since TGFβ1 is a potent regulator of cell proliferation and influences the size and shape of the limb [37]. We identified a QTL for the OC score at HH on SSC2 at 14 cM, while Christensen et al. [18] have reported QTL associated with cartilage thickening of the medial part of condylus humeri at 15 cM on the same chromosome. In addition, a QTL with dominance effect identified for the OC score at HH on SSC6 at 61 cM is located close to previously reported QTL for depression of the proximal edge of the radius at 51 cM [18] and for physis score at 75 cM [20]. QTL for HH on SSC3 at 13 cM and for CMH on SSC14 at 0 cM are new QTL (Figure 1). Interestingly, the QTL for CMF on SSC10 at 70 cM is close to a previously identified QTL regions at 75 cM for OC lesion in the subchondral bone of the medial part of condylus humeri and at 83 cM for fissure between cartilage and bone in pigs [18]. The QTL on SSC2 at 0 cM, close to marker *SW2443 *(SSC2p17), was the only QTL detected for BMC. One of the highest linkage associations, reaching a 5% GW significance, was found on SSC3 at 71 cM for BMD. A potential candidate gene in this chromosomal region is the follicle-stimulating hormone receptor (*FSHR*) gene, which directly regulates bone mass [15]. These QTL for BMC and BMD are novel and do not overlap with previously reported QTL.

Most of the identified QTL show large dominance effects rather than additive affects (Table 3). It is important to note that the transformation done on the leg score traits in this study did not change the identified QTL regions since the interval mapping results for these traits using the original score ranging from 1-5 or the scale 0-2 were the same. This implies that the transformation done on the leg score is not the reason for over-dominance in this experiment.

In another QTL study in the same population, 31 of 71 QTL for growth, fatness, leanness and meat quality traits have also shown high dominance effects [22], as well as QTL for immune traits [38]. Lee et al. [20] have also reported that most QTL for LW-related traits in a Large white × Meishan cross show dominance. In addition, using principal components analysis, Andersson-Eklund et al. [17] have identified a QTL for OC with a significant and large effect of over-dominance. Therefore, the results from this study and from previous studies reported in the literature [17-20,34] suggest that dominance plays a role in the genetic control of LW-related traits.

Most of the traits analysed in this study are categorical rather than normally distributed. Previous studies have shown that the QTL analysis method [39] used is suitable for categorical traits, with little loss of power [19,20]. The low heritability of these traits indicates that they may be complex traits and may be under a polygenic control primarily by non-additive gene action or affected by a major gene with Mendelian transmission [31]. In this study, most of the QTL were identified as single-trait regions. This could be explained by the low phenotypic correlations observed between the traits in the population.

## Conclusions

This is the first study identifying QTL affecting leg weakness and its related traits in a fast growing cross bred pig population between the Duroc and Pietrain breeds. Multiple QTL were detected for leg and feet scores, implying that these traits are controlled by multiple genes and that information from more than one QTL must be incorporated in selection procedures. Our results reveal novel QTL regions on SSC2 for BMC, on SSC3 for HH, on SSC6 and SSC18 for RLS, and on SSC14 for CMH, and also support some previously reported QTL regions. Although confidence intervals are large, these results will help to fine-map and identify candidate genes in these QTL regions using additional markers or gene polymorphisms located in the identified regions for LW-related traits in pigs.

## List of abbreviations used

ADG: average daily gain; BMD: bone mineral density; BMC: bone mineral content; BMA: bone mineral area; QTL: quantitative trait loci; DXA: dual energy X-ray absorptiometry; LW: leg weakness; FLS: fore leg score; RLS: rear leg score; FFS: fore feet score; RFS: rear feet score; OC: osteochondrosis; HH: head of the humerus; CMH: condylus medialis humeri; HF: head of the femur; CMF: condylus medialis femori; DuPi: Duroc × Pietrain resource population.

## Competing interests

The authors declare that they have no competing interests.

## Authors' contributions

WL performed OC phenotyping, analysed the phenotypes, prepared and drafted the manuscript. MU contributed to the data analyses, prepared and edited the manuscript. MC, CL and KW shared manuscript editing. CG calculated the genetic cards and helped with the statistical analysis. DT supervised the lab work. EJ and ET supervised the statistical analysis and edited the manuscript. AS analysed the DXA traits. HJ was responsible for animal breeding and for collecting leg and feet score phenotypes. HS and MM supervised the cartilage and bone collection and histological analyses of the OC trait. CP supervised the whole work and was included in project management and organisation of samples and work flow. KS supervised the study and edited the manuscript. All authors read and approved the final manuscript.

## Supplementary Material

Additional file 1 **Table S1 - Basis of scoring for legs, feet and osteochondrosis**. criteria used in this study to determine leg, feet and osteochondrosis scores Figure S1 - Sample of histological templates for the evaluation of OC score OC lesions are classified into four score values: (1) massive alterations of the cartilage including necrotic or ossified areas, (2) severe changes in the surface and deeper area of the articular cartilage like surface erosion, fibrillations, hyperplasia and chondrocyte necrosis, (3) cartilage shows few changes in surface and fibrillation, (4) cartilage surface is smooth, the matrix and chondrocytes are well organized with only a marginally rough surface or a weakly eosinophilic matrix or fibrillation Click here for file

Additional file 2 **Table S2 - Markers used in the QTL analysis and genetic map as established from the DuPi resource population**. ^a^numbers in brackets at the first and last marker are relative positions of those in the USDA-MARC v2 linkage map; ^b^S0226 not covered by USDA-MARC v2, but SW14, which is closely linked to S0226 (PigMap v 1.5); ^c^S0035 at 0 and S0003 at 144.5 cM in the International Workshop 1 SSC6 integrated map with a total length of 166.0 cM Click here for file

Additional file 3 **Table S3 - Analysis of variance for different LW-related traits**. ^1^FLS = fore leg score, RLS = rear leg score, FFS = fore feet score, RFS = rear feet score, OC = osteochondrosis, HH = head of the humerus, CMH = condylus medialis humeri, HF = head of the femur, CMF = condylus medialis femori, BMD = bone mineral density, BMC = bone mineral content, BMA = bone mineral area, ADG = average daily gain Click here for file
