# Supplementary material for: Quantitative trait loci analysis for leg weakness-related traits in a Duroc × Pietrain crossbred population
Source: Genet Sel Evol. 2011 Mar 20;43(1):13. doi: 10.1186/1297-9686-43-13 (PMC3072315; doi:10.1186/1297-9686-43-13)
Supplement: Additional file 1 — Table S1 - Basis of scoring for legs, feet and osteochondrosis. criteria used in this study to determine leg, feet and osteochondrosis scores Figure S1 - Sample of histological templates for the evaluation of OC score OC lesions are classified into four score values: (1) massive alterations of the cartilage including necrotic or ossified areas, (2) severe changes in the surface and deeper area of the articular cartilage like surface erosion, fibrillations, hyperplasia and chondrocyte necrosis, (3) cartilage shows few changes in surface and fibrillation, (4) cartilage surface is smooth, the matrix and chondrocytes are well organized with only a marginally rough surface or a weakly eosinophilic matrix or fibrillation [file 1297-9686-43-13-S1.PDF]

Table S1 - Basis of scoring for legs, feet and osteochondrosis.

Traits	Attribute	Scores				
		5	4	3	2	1
Legs	Strength of legs	Poor	Moderate	Very good	Moderate	Poor
	Straightness	Flexing	Slightly bent	Straight	Buckled	Very buckled
Feet				Scores		
				3	2	1
	Toes-soundness			Good	Moderate	Poor
	Toes-weight distribution			Even	Moderate	Uneven
	Angle of foot attachment			Good	Moderate	Low
	Damage			None	Slight or temporary	Permanent
OC			Scores			
			4	3	2	1
	Thickening of cartilage		Thick	Moderate	Thin	Very thin
	Cartilage structure		Good	Moderate	Low degradation and sporadic focal lesions	Degradation and focal lesions
	Vessels and cartilage canals structures		Good	Moderate	Low damage of cartilage canals	Damage vessels and cartilage canals

Figure S1 - Sample of histological templates for the evaluation of OC score

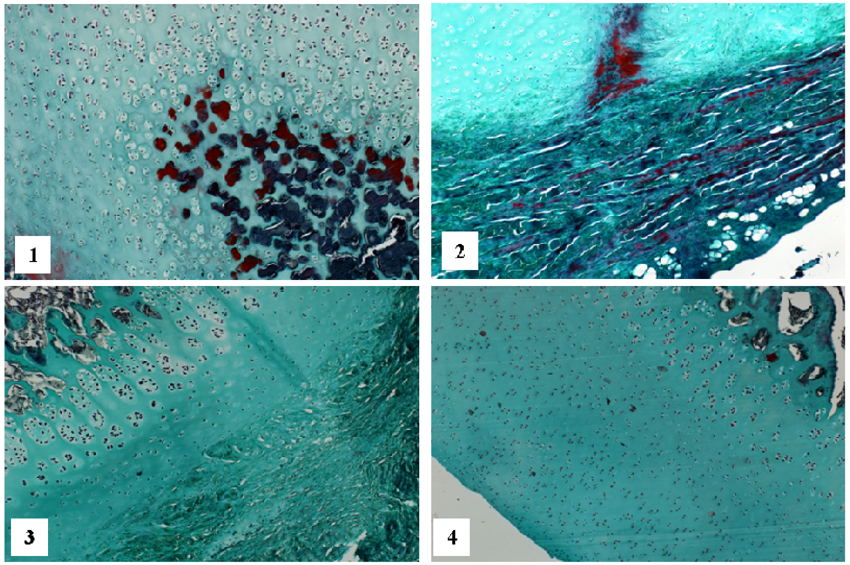

OC lesions are classified into four score values: (1) massive alterations of the cartilage including necrotic or ossified areas, (2) severe changes in the surface and deeper area of the articular cartilage like surface erosion, fibrillations, hyperplasia and chondrocyte necrosis, (3) cartilage shows few changes in surface and fibrillation, (4) cartilage surface is smooth, the matrix and chondrocytes are well organized with only a marginally rough surface or a weakly eosinophilic matrix or fibrillation
