# Supplementary material for: Quantitative trait loci analysis for leg weakness-related traits in a Duroc × Pietrain crossbred population
Source: Genet Sel Evol. 2011 Mar 20;43(1):13. doi: 10.1186/1297-9686-43-13 (PMC3072315; doi:10.1186/1297-9686-43-13)
Supplement: Additional file 2 — Table S2 - Markers used in the QTL analysis and genetic map as established from the DuPi resource population. anumbers in brackets at the first and last marker are relative positions of those in the USDA-MARC v2 linkage map; bS0226 not covered by USDA-MARC v2, but SW14, which is closely linked to S0226 (PigMap v 1.5); cS0035 at 0 and S0003 at 144.5 cM in the International Workshop 1 SSC6 integrated map with a total length of 166.0 cM [file 1297-9686-43-13-S2.PDF]

Table S2 - Markers used in the QTL analysis and genetic map as established from the DuPi resource population.

Chromosome	Markers and genetic distancesa (cM) [a]								
SSC1	SW1515(16.4)	34.8	SW1581	71.7	COL10A1	59.9	S0155	55.4	SW1301(140.5)
SSC2	SW2443 (0)	58.2	SW240	28.9	SW834	8.3	SW1517	8.3	S0226 (74.8)[b]
SSC3	SW72 (17.8)	33.5	S0164	26	SW2570	36.6	S0002(102.2)		
SSC4	S0227 (4.1)	50	S0001	31	S0214	49.3	S0097(120.0)		
SSC5	ACR (0)	10.9	SW413	31.9	SW1482	20.7	SWR453	14.2	
	S0092	16.9	S0005	41.5	SW1987	24.4	IGF1	48.7	SW967 (145.9)
SSC6	S0035 (7.3)[c]	61.2	S0087	13	SW1067	12.7	SW193	12.5	
	S0300	14.5	S0220	19.4	S0059	16.9	S0003 (102)[c]		
SSC7	S0025 (3.7)	33	S0064	36.6	S0102	16.9	SW175	31.5	
	S0115	38.9	S0101(134.9)						
SSC8	SW2611 (2.5)	89.6	S0086	27.6	S0144	12.8	SW61(112.3)		
SSC9	SW21 (11.1)	52.9	MMP3	50	SW911	23.4	SW54	15.1	
	S0109	25.6	S0295(96.5)						
SSC10	SW830 (0)	70.5	S0070	28.1	SWR67(122)				
SSC11	SW2008(14.1)	43.3	S0071	24.6	S0009	27.2	SW703(76.2)		
SSC12	SW2490 (0)	75.5	SW874	100	SW605(108.3)				
SSC13	S0219 (1.6)	44	SW344	37.4	SW398	87	S0289(112.1)		
SSC14	SW857 (7.4)	42.5	S0007	100	SWC27(111.5)				
SSC15	S0355 (1.3)	37.8	SW1111	47.8	SW936	40.8	SW1119(107.4)		
SSC16	S0111 (0)	67.2	S0026	89.1	S0061 (92.6)				
SSC17	SW335 (0)	40.4	SW840	99.6	SW2431(94.0)				
SSC18	SW1808 (0)	9.5	SW1023	70.9	SW787	40.3	SWR414 (57.6)		

[a]Numbers in the parentheses at the first and last marker are relative positions of those in the USDA-MARC v2 linkage map; [b]S0226 not covered by USDA-MARC v2, but SW14, which is closely linked to S0226 (PigMap v 1.5); [c]S0035 at 0 and S0003 at 144.5 in the International Workshop 1 SSC6 integrated map with a total length of 166.0.
