# Supplementary material for: Quantitative trait loci analysis for leg weakness-related traits in a Duroc × Pietrain crossbred population
Source: Genet Sel Evol. 2011 Mar 20;43(1):13. doi: 10.1186/1297-9686-43-13 (PMC3072315; doi:10.1186/1297-9686-43-13)
Supplement: Additional file 3 — Table S3 - Analysis of variance for different LW-related traits. 1FLS = fore leg score, RLS = rear leg score, FFS = fore feet score, RFS = rear feet score, OC = osteochondrosis, HH = head of the humerus, CMH = condylus medialis humeri, HF = head of the femur, CMF = condylus medialis femori, BMD = bone mineral density, BMC = bone mineral content, BMA = bone mineral area, ADG = average daily gain [file 1297-9686-43-13-S3.PDF]

Table S3 - Analysis of variance for different leg weakness-related traits.

Traits[1]		n	Mean±SE	Min/max	Model	R^2	Dam	Sire	Age	ADG	Slaughter weight	Carcass length	Parity
Leg score	FLS	310	2.65±0.94	1/5	***	0.38	***	*	ns	**	ns	*	***
	RLS	310	3.17±0.55	1/5	*	0.02	ns	ns	ns	*	ns	ns	ns
Feet score	FFS	310	2.02±0.44	1/3	**	0.18	*	ns	ns	ns	ns	ns	*
	RFS	310	2.53±0.48	1/3	***	0.21	***	ns	ns	ns	ns	ns	ns
OC score	HH	278	1.78±0.74	1/4	**	0.21	*	ns	ns	ns	ns	ns	**
	CMH	279	1.82±0.89	1/4	***	0.24	**	ns	ns	ns	ns	ns	***
	HF	274	1.98±0.81	1/4	*	0.20	*	ns	**	ns	ns	ns	***
	CMF	277	2.59±1.08	1/4	*	0.02	*	ns	ns	ns	ns	ns	ns
DXA	BMD	275	0.96±0.07	0.69/1.25	***	0.42	*	ns	ns	ns	***	ns	***
	BMC	275	66.72±4.31	45.53/87.36	***	0.68	***	ns	ns	ns	***	***	**
	BMA	275	69.67±3.86	55.91/84.64	***	0.54	***	ns	ns	ns	***	***	**

[1]FLS=fore leg score, RLS=rear leg score, FFS=fore feet score, RFS=rear feet score, OC=osteochondrosis HH=head of humerous, CMH=condylus medialis humeri, HF=head of femur, CMF=condylus medialis femori, DXA= dual energy X-ray absorptiometry, BMD=bone mineral density, BMC=bone mineral content, BMA=bone mineral area, ADG= average daily gain
